# Molecular Mechanisms behind Conformational Transitions of the Influenza Virus Hemagglutinin Membrane Anchor

**DOI:** 10.1021/acs.jpcb.3c05257

**Published:** 2023-10-25

**Authors:** Michal Michalski, Piotr Setny

**Affiliations:** Centre of New Technologies, University of Warsaw, 02-097 Warsaw, Poland

## Abstract

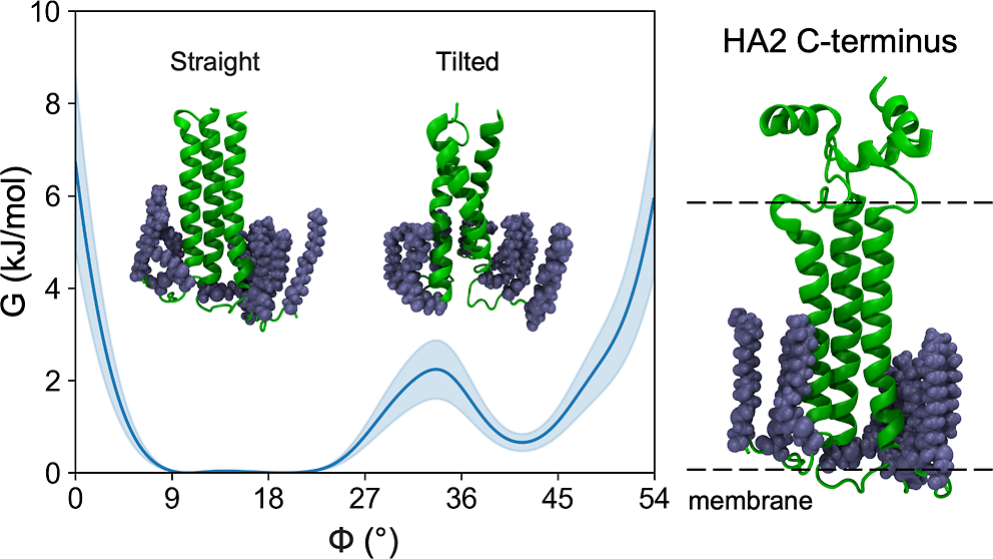

Membrane fusion is a fundamental process that is exploited by enveloped viruses to enter host cells. In the case of the influenza virus, fusion is facilitated by the trimeric viral hemagglutinin protein (HA). So far, major focus has been put on its N-terminal fusion peptides, which are directly responsible for fusion initiation. A growing body of evidence points also to a significant functional role of the HA C-terminal domain, which however remains incompletely understood. Our computational study aimed to elucidate the structural and functional interdependencies within the HA C-terminal region encompassing the transmembrane domain (TMD) and the cytoplasmic tail (CT). In particular, we were interested in the conformational shift of the TMD in response to varying cholesterol concentration in the viral membrane and in its modulation by the presence of CT. Using free-energy calculations based on atomistic molecular dynamics simulations, we characterized transitions between straight and tilted metastable TMD configurations under varying conditions. We found that the presence of CT is essential for achieving a stable, highly tilted TMD configuration. As we demonstrate, such a configuration of HA membrane anchor likely supports the tilting motion of its ectodomain, which needs to be executed during membrane fusion. This finding highlights the functional role of, so far, the relatively overlooked CT region.

## Introduction

Influenza is a respiratory system disease that humanity has been facing since ancient times.^1^ Currently, it is estimated to affect 10–20% of the global population every year, causing a significant number of deaths despite a relatively low mortality rate. One of the major challenges posed by influenza is its high mutation rate, which constantly threatens the possibility of a pandemic. The virus responsible for influenza is enveloped and primarily spreads through respiratory droplets. It enters host cells via an endocytosis process, and the final step in this process involves the fusion of viral and endosomal membranes, allowing the release of infectious genetic material into the cytoplasm.^2^

The structure of the influenza A virus is usually spherical or ovoid in shape with 80 to 120 nm in diameter.^3^ The two most abundant proteins on the surface of influenza virus particles are hemagglutinin (HA) and neuraminidase. The HA is a homotrimeric protein, which is anchored within a viral membrane. It plays a significant role in influenza virus entry into the host cell, by initiating the fusion of viral and endosomal lipid membranes.^4^ The HA consists of two subunits: HA1, which is responsible for virus binding to cell receptors, and HA2, which controls the actual fusion process.^5^ Based on the amino acid sequence, as well as on the 3D structures of their ectodomains, the 18 antigenic subtypes of HA are divided into two main phylogenetic group-1 and group-2 groups.^6−8^

The HA plays multiple roles in viral entry into a host cell. First, HA binds to the sialic acid found on glycoproteins or glycolipid receptors of the host membrane, which initiates the endocytosis process. Second, once the pH in an endosome is lowered, HA undergoes partial refolding which results in the formation of an extended coiled coil stem structure directed toward the endosomal membrane.^9^ Third, HA releases fusion domains and inserts them into the endosomal membrane.^10^ Being anchored in the viral membrane through the C-terminal transmembrane domains (TMDs)^11^ and cytoplasmic tails (CTs)^12^ and in the endosomal membrane through the N-terminal fusion domains,^13,14^ HA executes a jack-knife motion and brings the two membranes into close contact. It allows overcoming the dehydration barrier and ultimately leads to the formation of a stalk structure, which serves as the first fusion intermediate.^15,16^ Notably, in the course of this process, rigid parts of the ectodomain need to tilt significantly with respect to their membrane anchors to fit between the apposing membranes.

While the ectodomain 3-D structure has been resolved using X-ray diffraction,^17,18^ and almost complete HA, including the N-terminal part of the TMD, was recently determined by cryo-EM, the structure of the C-terminal part of the TMD as well as of the CT remain entirely unknown.^11,19−21^ The TMD is composed of 26–27 amino acid residues, and its N-terminal part that contacts the outer half of the lipid bilayer is highly conserved, both within group-1 and group-2 HAs. A high sequence conservation suggests that the N-terminal domain not only is a membrane anchor but also might play more specific role, for example, binding to hydrophobic ligands. Current studies identify a conserved cholesterol recognition motif in group-2 HAs that includes a completely conserved tyrosine and a lysine in the linker region and a leucine and a tryptophan at the beginning of the TMD, yet its relevance remains to be confirmed.^22^ A 30 residue peptide including the TMD of influenza strain (group-2 HA) was found to be predominantly α-helical in detergent micelles and in phospholipid bilayers, as assessed by circular dichroism (CD) and attenuated total reflection Fourier transform infrared spectroscopy.^19^ Molecular dynamics (MD) simulation of a 27 residue TMD peptide from group-1 HA confirmed a mostly α-helical, tilted structure when embedded within a phospholipid bilayer. Once three copies of the peptide were incorporated into the bilayer, they formed a triangular, parallel assembly thought to resemble the structure of native trimeric HA.^12,23^

**Figure 1:**
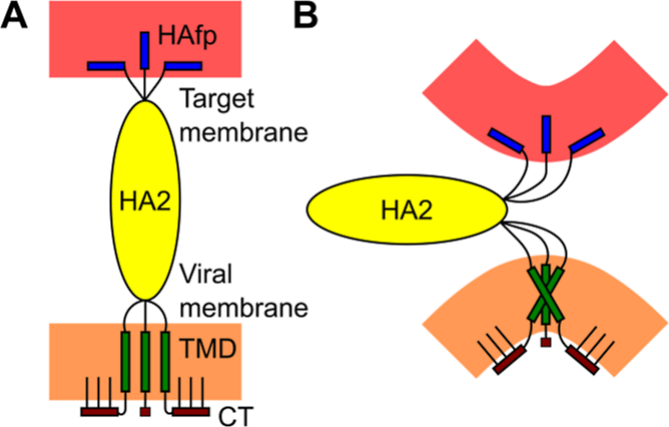
Schematic representation of the putative TMD rearrangement during the ectodomain tilting motion. (A) Extended structure prior to membranes apposition and (B) tilted structure in the prefusion state. HAfp—fusion peptide, HA2—ectodomain, and TMD—transmembrane domain.

Recent structural studies have shown that the TMD region of HA is involved in ectodomain tilting with respect to the membrane surface.^11^ Interestingly, in addition to variations in chain orientation within the flexible linker connecting the ectodomain to the TMD, the three TMD helices alone rotate relative to each other in the tilted HA trimers. The analysis of intermolecular links between transmembrane helices reveals the participation of tyrosine residues at position 190, suggesting that this amino acid is involved in maintaining the integrity of the helical bundle at different tilt angles.^11^ The rearrangement of the membrane-associated region may facilitate pH-dependent changes in HA conformation, which are required for membrane fusion, and finally support the tilting motion of the HA ectodomain in the narrowing space between the viral and cellular membranes as they are brought into contact for fusion (Figure 1).^24^ The importance of TMD structural changes during HA tilting motion remains to be verified, and particularly interesting in this context is the dependence of TMD conformational equilibrium on the membrane composition.

The lipid composition of the viral membranes is determined by the composition of the cell membrane at which the viruses are assembled and released by budding. The influenza viral membrane appears to be enriched in cholesterol (CHL) and sphingolipid,^25^ but detergent micelles used in experimental measurements differ from the native environment of the HA transmembrane region and were only selected for their ability to preserve the structure of membrane proteins.^26,27^ Particularly intriguing is the role of the CHL content in the viral membrane. It is known that HA clusters in a CHL-dependent manner on the plasma membrane of transfected cells,^28^ which one may suppose is a consequence of hydrophobic mismatch between the length of the transmembrane domains and the thickness of the lipid membrane.^29,30^ It is also known that CHL increases the fusion propensity of phosphatidylcholine (PC)/phosphatidylethanolamine (PE) membranes^31,32^ and stabilizes negatively curved stalk regions.^33^ One postulated mechanism is related to its presence in the viral membrane and interaction with HA TMD, which is supposed to promote HA partitioning into the fusion region.^34,35^ The above observations raise a question concerning the actual role of specific HA TMD–cholesterol interactions, including the mechanism of cholesterol sensing by TMD, and furthermore the role of those interactions in HA tilting motion occurring in the course of the fusion event.

The CT is a short 11–12 amino acid and a highly conserved peptide. Intriguingly, it possesses five residues that are almost invariant, not only within one group but also through all HA subtypes.^36,37^ Such high conservation of those amino acids suggests functional importance. Three C-terminal CT residues form a highly hydrophobic patch. A completely conserved glycine is observed at position 214, which might have a substantial influence on the CT secondary structure and orientation relative to that of the viral membrane. The CT contains two cysteines strictly conserved through all HA variants, which are post-translationally palmitoylated, and one highly conserved cysteine at the border with the TMD, which is predominantly acylated with stearate.^36,38^ Interestingly, if HA subtype does not have a cysteine at one of the positions, it is replaced with a hydrophobic amino acid, which suggests that these positions have the propensity to interact with lipid membranes.^39^ While the exact function of CT is still unknown, most recent studies suggest that the entire C-terminal region of HA does not function simply as a membrane anchor, but TMD, CT, and especially the covalently bound fatty acids might be crucial for raft localization of the trimeric HA, for virus assembly, and for opening and widening of the fusion pore during viral entry.^40−45^

In addition to unresolved questions concerning the possible functional role of HA CT, little is known about its 3-D structure. In an experimental study, CT was postulated to exist as a β-strand peptide.^46^ However, the study suffered from significant limitations: (i) the secondary structure was postulated based on synthetic peptides in the monomeric state, (ii) in a water environment (at pH = 7.4 and pH = 5.0), and (iii) without S-acylation. It is thus still unclear what kind of secondary structure CT forms. One may assume that the reason the structure of this domain has yet to be obtained is not because of the intrinsically disordered nature of this region but mostly because of its complicated configuration in relation to the lipid membrane. To take into account this phenomenon, fatty acids are anchored to the respective membrane and bound to the cysteine residues via thioester bonds.^47,48^ It is almost impossible to reproduce the configuration of such a complex system in vitro. The NMR technique cannot be used to get a reliable structure due to strong tendency of this peptide to aggregate within membrane mimetic environments.^12^ Interestingly, in another study, different patterns of S-acylation of the HA2 C-terminal anchoring segment were proposed to contribute to fine-tuning of TMD homotrimer packaging and stabilization.^49^ Finally, it has not been verified whether the β-strand conformation of CT trimers proposed for monomeric states is actually possible under constraints imposed by the TMD length, structure, and requirement to sustain ectodomain tilting. Linking structural insights into CT with the analysis of CHL-dependent TMD configurations, with and without its C-terminal tail, is essential to explain possible fine-tuning and stabilization of TMD, and ultimately, the observed dependence between fusion activity and CT amino acid sequence.^50^

In the current work, we use fully atomistic MD simulations to investigate TMD configurations and CT structure in a viral-like lipid bilayer. First, we perform unrestrained MD to explore metastable TMD states and the structure of CT under a native, trimeric arrangement and with specific cysteine residues modified by palmitoylation. Second, we carry out umbrella sampling simulations to calculate the potential of mean force (PMF) for the TMD transition between straight and tilted states under different conditions to assess CHL and CT influence on this process. Lastly, we conduct a set of simulations to investigate TMD-dependent tilting of the HA ectodomain fragment in order to capture the possible impact on the fusion mechanism.

## Materials and Methods

**Figure 2:**
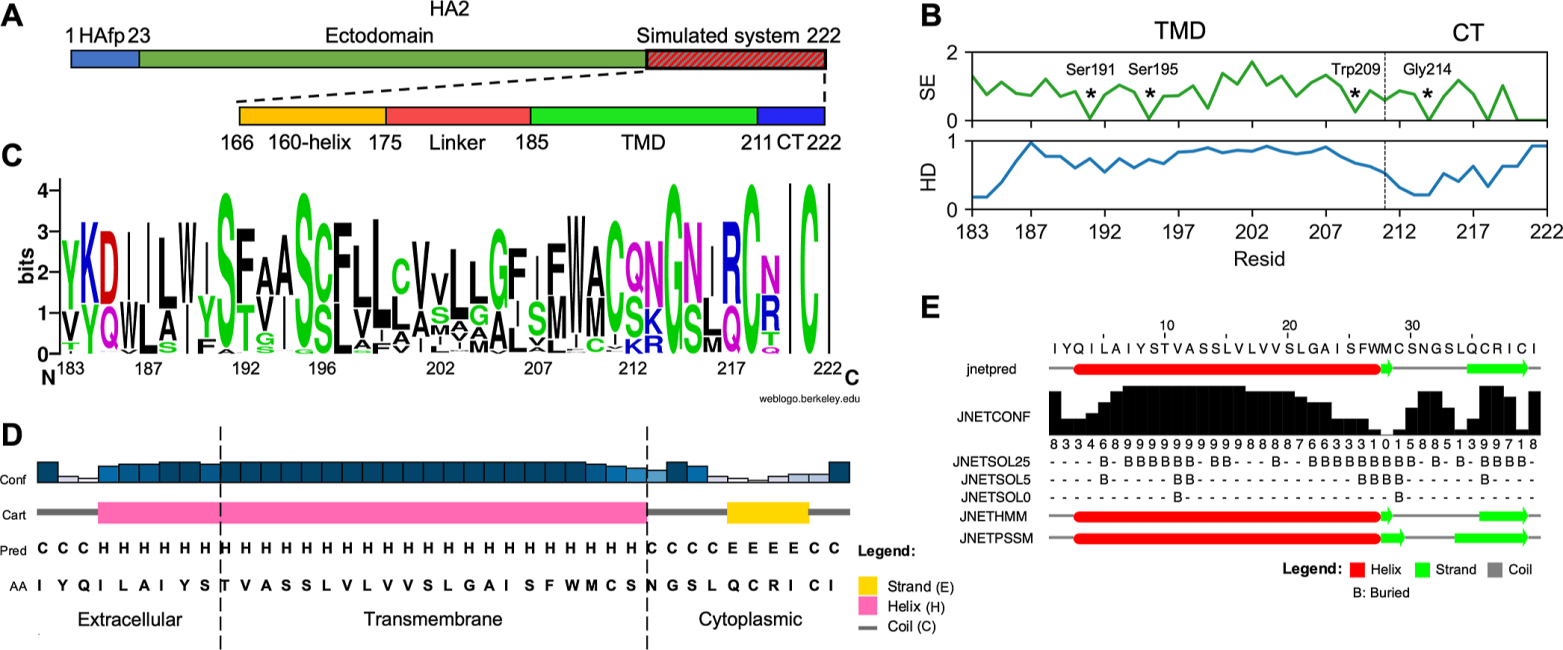
(A) Schematic representation of the influenza virus HA2 subunit with focus on the C-terminus region. (B) Shannon entropy (SE) and hydrophobicity (HD) normalized to span the [0,1] range, along with increasing hydrophobic character. (C) Sequence conservation. (D) Membrane localization and secondary structure prediction with confidence assessment (Conf) by PSIPRED 4.0. (E) Secondary structure and residue burial predictions by JPred 4.

We used MD simulations to explore TMD and CT configurations in lipid membrane with and without CHL. The straight and tilted TMD configurations were modeled (Supporting Information Figure S1) based on cryo-electron microscopy (cryo-EM) structures with PDB IDs 6HJQ and 6HJR, respectively. Missing protein parts, including the C-terminal part of TMD (residues 204–210) and CT (residues 211–222), were reconstructed using the BIOVIA Discovery Studio 2021.^11,51^ We assumed α-helical conformation for the entire TMD (in accordance with the expected geometry, confirmed also by bioinformatic analysis—see Figure 2D) and an extended structure for the CT fragment (such that to avoid any preliminary bias). Protonation states of titratable residues were checked using the PropKa server.^52−54^ Since no p*K* was found within the range 5–7, relevant for hemagglutinin functioning, we kept all amino acids in their standard forms. The structures included post-translational palmitoylation for cysteine residues within CT fragments.

The TMD was embedded in a viral-like membrane comprising 32 (13.3%) POPC, 56 (23.3%) POPE, 44 (18.3%) POPS, and 108 (45.0%) CHL molecules.^55^ In addition, a CHL-free bilayer composed of 52 (21.6%) POPC, 108 (45.0%) POPE, and 80 (33.3%) POPS lipids was considered. The protein–membrane system was solvated in aqueous solvent with 150 mmol/L Na^+^ and Cl^–^ ions plus excess ions necessary to neutralize the total charge. The system was assembled using the CHARMM-GUI server.^56^ Proteins, lipids, and ions were parametrized with the CHARMM36M force field,^57^ and the TIP3P model^58^ was used for water. During simulations, covalent bonds to hydrogen atoms were constrained using the LINCS method,^59^ and a 2 fs time step was employed. Electrostatic interactions were treated with the use of the particle mesh Ewald method,^60^ and 1.2 nm cutoff for Lennard–Jones potential with a force switch smoothing function from 10 to 12 Å was applied. The simulations were run at a temperature of 310 K, controlled by the Nosé–Hoover thermostat,^61^ and at a pressure of 1 bar, maintained by a semi-isotropic Parrinello–Rahman barostat.^62^ All simulations were conducted with the GROMACS software,^63^ with the standard CHARMM-GUI protocol used for system equilibration.

The potentials of mean force (PMFs) for TMD transitions between straight and tilted geometries were calculated using the umbrella sampling method. A collective variable, ϕ, was introduced as the mean of three pairwise angles between three TMD α-helix axes, defined by vectors extending between Ile189-Thr192 and Ser207-Met210 Cα atoms. For each system, we performed a preliminary slow growth simulation for 100 ns, gradually changing ϕ from 0° (ideally straight configuration) to 55° (extensively tilted configuration) at temperature *T* = 310 K, using *V*(ϕ) with a force constant *k* = 3000 kJ mol^–1^ rad^–2^. Each system was sampled along 19 windows, evenly spaced across the ϕ range, using a harmonic biasing potential with a force constant *k* = 3000 kJ mol^–1^ rad^–2^. Each production window of the umbrella sampling simulations was sampled for a minimum of 500 ns following 500 ns equilibration. The calculation of ϕ and the application of the biasing potential were handled by the PLUMED 2.5.1 module.^64^ The data was processed using the weighted histogram analysis method (WHAM),^65^ with bootstrap error analysis, as implemented in GROMACS.

Unrestrained 3 μs MD simulations of TMD-CT systems in straight and tilted configurations were utilized to verify the stability of experimentally derived TMD configurations and to examine the respective CT conformations in the membrane-bound state. The analysis of the latter was based on the root-mean-square deviation (RMSD) of CT Cα atoms. The RMSD matrices were constructed for concatenated CT trajectories sourced from both MD runs, resulting in a total simulation time of 18 μs. The β angle, which represents the degree of CT bending, was calculated between Cα atoms of Ser212, Gln217, and Cys221 residues. Clustering analysis was executed using the GROMOS method, implementing the GROMACS gmx cluster tool.^63^ The optimal cluster number was determined through a visual dendrogram inspection.

To investigate the coupling between the TMD configuration and the HA ectodomain tilt, ten independent 3 μs MD simulations were conducted to represent five straight and five tilted TMD geometries, respectively. The ectodomain tilt with respect to membrane was characterized by an angle, θ, between the vector orthogonal to the plane defined by Glu171 Cα atoms of the three α-helices representing the HA ectodomain 160-helix (residues 160–174)^11^ and the membrane normal. The 160-helix was connected with TMD by the linker domain (residues 175–184). Both domains were obtained from the cryo-EM structure (PDB: 6HJQ).^11^ In a starting structure for each simulation, the ectodomain was modeled with θ = 0. In order to conserve the 160-helix assembly, simulations were conducted with harmonic restraints, with a force constant of 1000 kJ mol^–1^ nm^–2^ and reference distances, obtained from the cryo-EM structure (PDB: 6HJQ), between Ser166 and Asp177 Cα atoms (Supporting Information Table S1).

Bioinformatic analysis of HA TMD and CT domains involved the alignment of C-terminal amino acid sequences from the UniProt database^66^ using the BLAST algorithm.^67^ The WebLogo 3.3 server^68^ was used to illustrate sequence variability. Shannon sequence entropy was evaluated through the Los Alamos National Laboratory HIV Sequence Database^69^ software. Hydrophobicity (HD) analysis was performed based on the Kyte and Doolittle scale via the ProtScale web service.^70,71^ Secondary structure predictions were carried out by PSIPRED 4.0 and JPred 4 tools.^72,73^ The hydrophobic length of TMD was assessed using the PPM 3.0 module within the OPM web server^74,75^ based on calculations for 3 simulation snapshots per S and T states. Membrane hydrophobic thickness was evaluated based on additional 300 ns long protein-free simulations of considered membranes. It was calculated using the density distribution of lipid CH_2_ groups as a distance between points corresponding to half of maximal densities on both membrane sides.^76^

## Results and Discussion

### Bioinformatic Analysis

In the current study, we aimed to elucidate structural characteristics of the HA C-terminal segment, which encompasses TMD and CT domains (see Figure 2A). Despite the vital role of this membrane-anchored HA fragment in supporting HA rearrangements during the fusion process, our understanding of its structural plasticity remains limited. Bioinformatic analysis confirms that the TMD sequence supports α-helical conformation and is of highly hydrophobic character,^11,77,78^ typical for membrane-spanning protein domains.^11^ These features are highly preserved across all HA variants, indicating a high level of specialization. Of note is that there is near absolute conservation of two serine residues (Ser191 and Ser195) at the N-terminal TMD segment and a tryptophan residue (Trp209) at the C-terminal part. Whereas the presence of tryptophan is typical at the interface regions of TMDs,^79^ the occurrence of buried serine residues, which possess relatively polar side chains, might indicate a specific functional role. One possibility is their involvement in the stabilization of the symmetric arrangement of three TMD α-helices. Such a symmetric arrangement, which is also suggested by experimental studies, would be a natural consequence of the overall HA geometry, at least in a prefusion state in which the ectodomain is perpendicular to the viral surface.

The CT segment is relatively short (11 residues) and rich in hydrophobic amino acids (Figure 2B). It contains a few residues whose absolute or almost absolute conservation (Figure 2C) across different HA variants (Figure 2C) may indicate a critical functional role. Three of them are cysteines, which are known to undergo post-translational palmitoylation. They increase CT hydrophobicity and facilitate protein trafficking to cellular membranes.^80,81^ Another conserved residue is Gly214, whose presence may indicate a propensity of the CT structure to form a bend at this position. Indeed, secondary structure prediction models propose a β-sheet or bent conformation for CT (see Figure 2D,E); however, the confidence level of those predictions is limited. To this end, it is essential to recognize the limitations of such models, as they do not account for the influence of the cellular environment at the water–membrane interface and neither do they consider the effect of post-translational modifications like palmitoylation, which may further impact the function and localization of the CT segment.

### TMD Configurations

**Figure 3:**
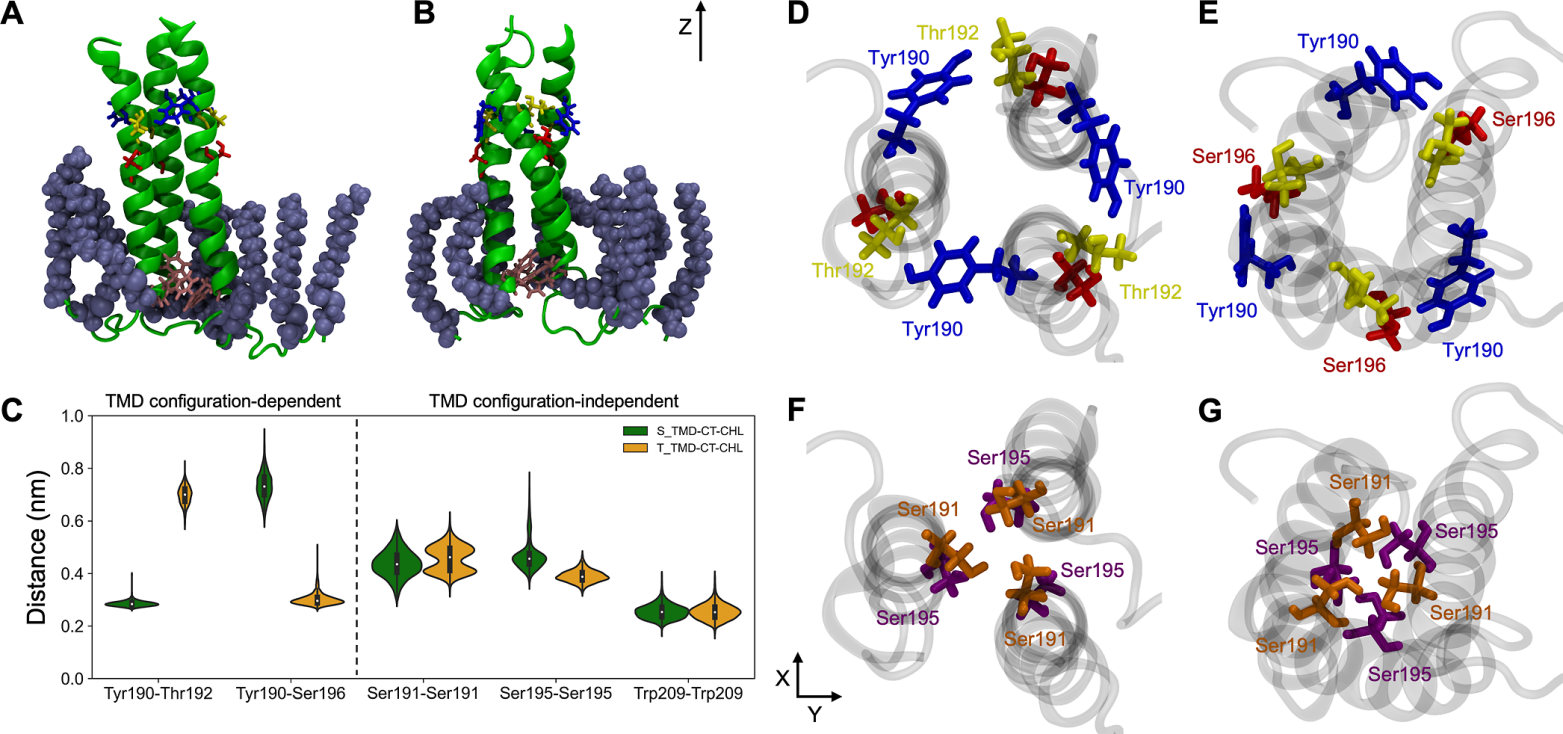
(A,B) Straight and tilted TMD configurations, respectively, after 3 μs of simulation time. (C) Distributions of donor–acceptor distances for hydrogen bonds calculated for Tyr190:OH···Thr192:OG1, Tyr190:OH···Ser196:OG, Ser191:OG···Ser191:OG, Ser195:OG···Ser195:OG, and for minimal distances between Trp209 heavy atoms within TMD for the last 1 μs of simulation time. (D–G) Main interactions in the TMD N-terminus stabilizing both configurations.

Recent cryo-EM studies revealed two distinct HA TMD configurations (see Figure 3A,B).^11^ The first one, referred to as the ”straight”, S, state, is characterized by a roughly parallel arrangement of the 3 membrane-spanning α-helices. The second configuration, termed the ”tilted”, T, state, exhibits a noticeable rotational shift of the helix axes relative to each other. Both configurations are stabilized by intermolecular links, among which a significant role is seemingly played by tyrosine residues.^11^ In order to further investigate these distinct structural arrangements, we performed unrestrained MD simulations of trimeric TMD with palmitoylated CTs in the S and T states, embedded in a viral-like membrane. Each variant was independently simulated for 3 μs, of which the final 1 μs was used for analysis.

The membrane-bound proteins in both states did not exhibit significant departures from their corresponding starting geometries during MD simulations, confirming the relevance and stability of two diverse, experimentally obtained TMD configurations (Supporting Information Figure S2). The analysis of prevalent interhelix interactions revealed the presence of three distinct rings of specific residue contacts at the N and C termini of the TMD α-helices (Figure 3A–G). Notably, the formation of the ring involving Tyr190 turns out to occur in a TMD configuration-dependent manner and relies on the hydrogen-bonding capabilities of the tyrosine side chain. In the S TMD state, Tyr190 hydroxyl groups form stable bonds (donor–acceptor distance in the order of 0.3 nm) with hydroxyl groups of Thr192 from adjacent helices. However, upon TMD transition to the T state, these interactions are broken, and Tyr190 side chains are rotated deeper toward the membrane core where they form hydrogen bonds with hydroxyl groups of Ser196. Remarkably, the rearrangement of hydrogen bonds, which involves ∼0.5 nm difference in the acceptor–donor distances, occurs symmetrically at all three interfaces between TMD helices. The second specific set of interactions identified at the N-terminal part is formed between Ser191 and Ser195 residues and is independent of the TMD configuration. Both of these residues display a high degree of conservation across all HA types (see Figure 2B), what suggests a likely evolutionary pressure toward maintaining these contacts.

The interaction ring at the C-terminal TMD segment is formed between highly conserved Trp209 residues. The localization of tryptophan near the membrane–water interface is a characteristic feature of transmembrane proteins. These residues act as atomistic anchors guiding the proper insertion of transmembrane segments, contribute to fine adjustments in their orientation, and also serve as landmarks at helix–helix interfaces.^79,82,83^ In our simulations, regardless of the TMD configuration, we observed the formation of stable contacts, defined as the minimal distance between heavy atoms below 0.3 nm, through indole rings, which likely facilitate the proper arrangement and structural integrity of the TMD at the internal viral side.

The analysis of lipid distribution in the immediate proximity of TMD structures does not reveal any specific lipid binding, in particular involving CHL molecules. In fact, CHL seems to be slightly depleted around TMD within ∼1 nm range, in favor of negatively charged POPS lipids (Figure S3). This phenomenon can be attributed to the overall positive charge on the TMD, 3*e*, which arises from the presence of Arg217 residues within the CT regions. According to our simulations, POPS enrichment and CHL depletion are somewhat more pronounced around the T state, compared to the S state. Given, however, that the estimated hydrophobic lengths of both states are nearly identical (3.57 nm vs 3.56 nm, for S and T, respectively, Table S2) and fit very well to membrane hydrophobic thickness (3.60 nm, Table S3), it does not appear that protein–lipid interactions exert any discernible impact on the equilibrium between the S and T states.

### TMD Structural Transitions

The unrestrained MD runs discussed above indicate the metastable character of the S and T TMD configurations. The fact that we observed no spontaneous conversion between them suggests the existence of a free energy barrier that is prohibitively high to be efficiently crossed in the available simulation time. Accordingly, in order to investigate the barrier height, G^⧧^, as well as relative depths of the respective free energy basins, Δ*G*_T→S_, we conducted a series of umbrella sampling simulations aiming to obtain the potential of mean force between them. To this end, we introduced a reaction coordinate, ϕ, based on the average angle between the axes of TMD α-helices. Specifically, ϕ = 0° corresponds to the parallel alignment of the three α-helices and ϕ ∼ 50° to a highly tilted TMD state. For reference, in the unrestrained simulations of the S and T TMD-CT configurations, ϕ was observed to sample the region of ∼15°, and ∼40°, respectively (Supporting Information Figure S4).

In order to gain insights into the role of the CT segment as well as the presence of CHL within the membrane, we explicitly considered four distinct system setups: (1) TMD with CHL in the membrane, (2) TMD without CHL in the membrane, (3) TMD with attached CT in the presence of CHL, and (4) TMD with attached CT and without CHL in the lipid bilayer. We note, however, that the assumed model of the HA C-terminus does not consider the influence of the ectodomain part to allow for a more focused and detailed analysis of the TMD core transition, given the available computational resources.

**Figure 4:**
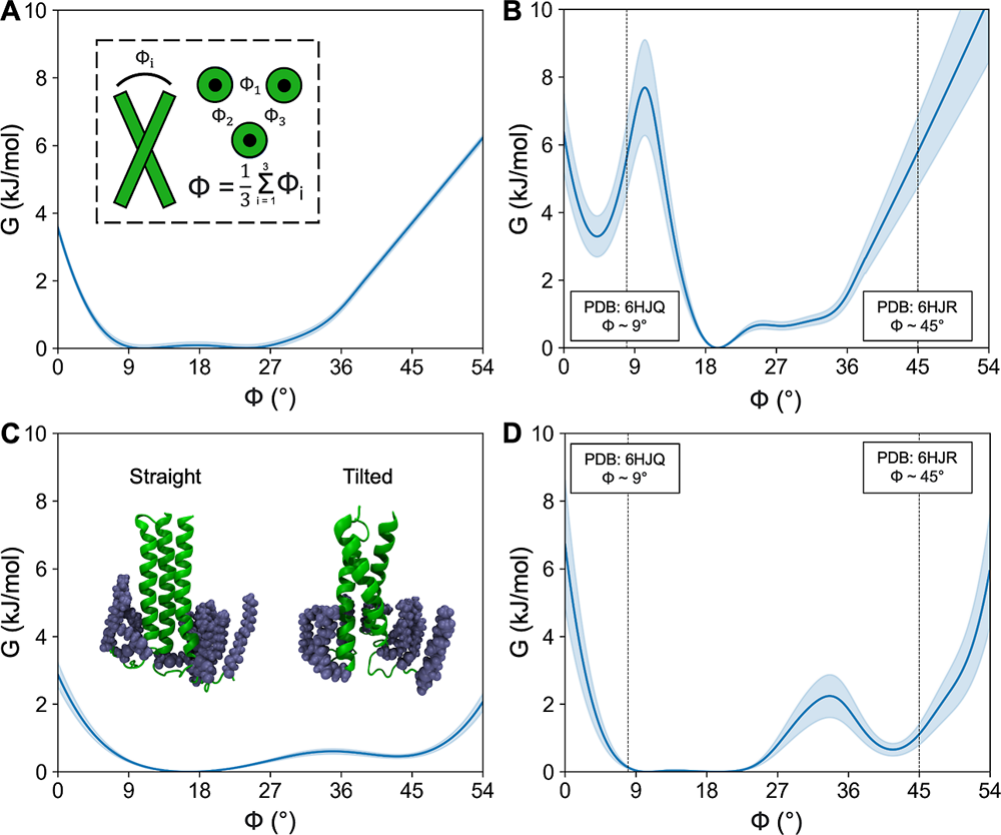
PMFs for TMD transition from straight to a tilted state in four system setups: (A) TMD without CT and membrane without CHL, (B) TMD without CT and membrane with CHL, (C) TMD with CT and membrane without CHL, and (D) TMD with CT and membrane with CHL. Vertical dashed lines indicate ϕ values from cryo-EM structures (PDB: 6HJQ and 6HJR). Blue shadows correspond to the bootstrapping error.

The PMF obtained for TMD without the CT segment and embedded in a membrane devoid of CHL (see Figure 4A) does not display the expected two minima, indicative of both S and T TMD configurations. Instead, a single broad minimum is observed. Apparently, this specific membrane composition, with an average P–P thickness of 4.25 nm (Supporting Information Figure S5, Table S3), effectively prevents CT-devoid TMD from adopting any distinct configuration. This is directly manifested by the instability of interactions formed by Tyr190, whose engagement in hydrogen bonds with either Thr192 or Ser196 would be expected for the stable S or T state, respectively (Supporting Information Figure S6).

The separation of the TMD conformational ensemble into two distinct, experimentally confirmed states is rescued by the introduction of CHL into the membrane (see Figure 4B). This change is associated with an increase in the bilayer P–P thickness to 4.5 nm. It is of note, however, that in the absence of the CT, TMD α-helices are closer to being parallel in the S state and do not reach a full tilt in the T state, compared to unrestrained simulations involving the complete HA C-terminus in a CHL-containing membrane. The free-energy minima are separated by G^⧧^ ∼ 8 kJ/mol for the T → S transition, and the PMF indicates Δ*G*_T→S_ in the order of 3 kJ/mol. The latter result is at odds with experimental studies, which demonstrated some preference for the S TMD state,^11^ and suggests a possibly important role of the CT in shaping the TMD conformational equilibria.

The impact of CT presence on the TMD conformational behavior is evident in simulations without and with CHL in the membrane (Figure 4C,D, respectively). In both scenarios, the simulations reveal the existence of free-energy minima located around ϕ values consistent with those observed in unrestrained simulations of the TMD-CT system in the presence of CHL. Importantly, the respective TMD configurations display distinct Tyr190 interaction patterns that are indicative of S and T states. The free energy barrier between S and T states is practically nonexistent in the absence of CHL, whereas it achieves ∼2 kJ/mol after its addition. Remarkably, the system that best approximates the native state, namely, with both CT and CHL present, shows a slight TMD preference toward the S state, in agreement with the experimental assessment. Taken together, these observations indicate that the CT exerts considerable influence on the equilibrium between the S and T states of the TMD. Furthermore, and perhaps more importantly, it appears to secure TMD stability over a broad range of interhelix angles, in particular, enabling the adoption of highly tilted T states.

**Figure 5:**
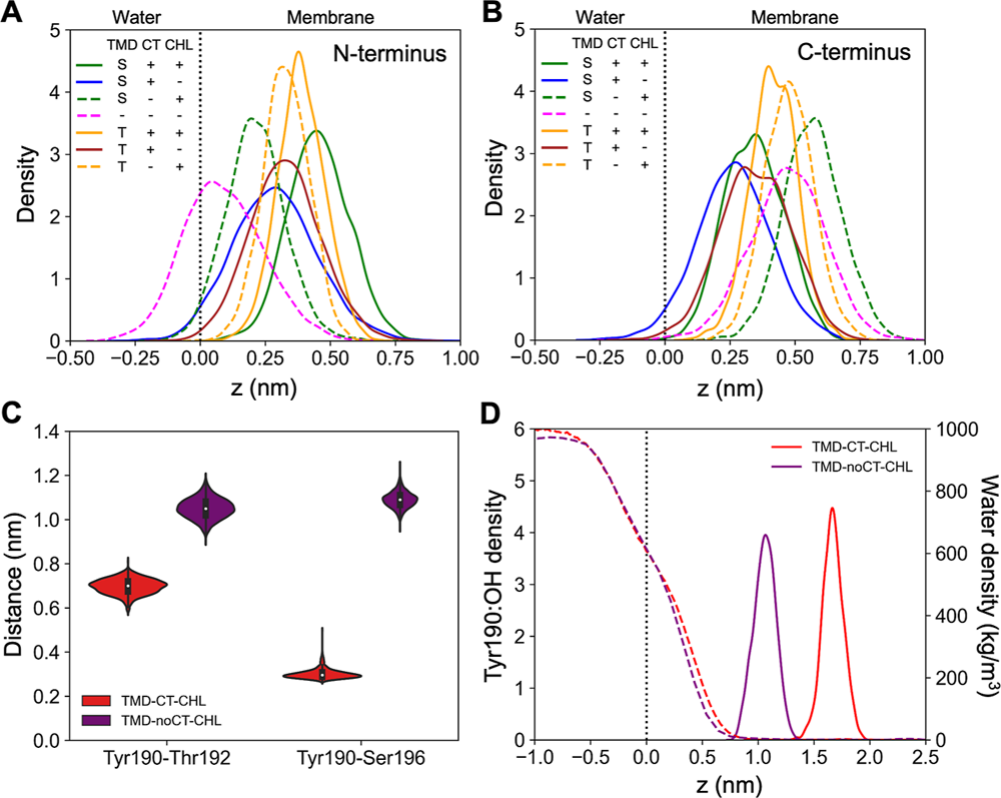
(A) Distribution of the TMD N-terminus (GLN185:Cα atom) position and (B) TMD C-terminus (CYS211:Cα atom) position along the membrane normal axis (*z*) with respect to the corresponding membrane–water interfaces (peak phosphate atom density, *z* = 0). (C) Donor–acceptor distances for hydrogen bonds between Tyr190:OH···Thr192:OG1 and Tyr190:OH···Ser196:OG for TMD kept at ϕ = 45°. (D) Distributions of Tyr190:OH atoms and water oxygen atoms along the *z* axis for TMD kept at ϕ = 45°.

To gain a physical understanding of the effects associated with the presence of the CT, we analyzed the location of N and C terminal TMD residues (Cα atoms of Gln185 and Cys211, respectively) relative to the membrane surface under the different considered scenarios (Figure 5A,B). The CT turned out to act as an anchor, firmly securing the burial of TMD C-terminus at the level ∼0.3 nm below the membrane surface, irrespective of TMD tilt and CHL presence (Figure 5B). In all cases, this phenomenon led to a deeper positioning of the TMD-CT N-terminus within the lipid bilayer, compared to the corresponding systems without the CT (Figure 5A).

Such a relocation is expected to stabilize hydrogen bonds within the N-terminal TMD interaction rings by moving them away from the aqueous solvent. A striking effect is observed in the case of Tyr190–Ser196 interaction that is supposed to form in the T state but remains broken in the absence of CT, even if the TMD is pulled to ϕ ∼ 45° by the biasing potential applied in umbrella sampling runs (Figure 5C). The analysis of the Tyr190 hydroxyl oxygen atom position along the membrane normal for the TMD held around ϕ ∼ 45° indicates its complete burial in the CT-containing system and relative exposure to the aqueous environment in the CT-free scenario (Figure 5D). It suggests that Tyr190 fails to engage in an interaction with Ser196 due to favorable competition for hydrogen bonds of water molecules.

In light of the above findings, we draw a conclusion that CT induces a specific localization of the N-terminal TMD hydrogen bond rings, positioning them deeper within the hydrophobic lipid environment. Their resulting shielding from the aqueous environment not only enhances TMD stability but also provides for effective switching of the interaction pattern between two distinct TMD states. This mechanism likely underscores the HA conformational adaptability, enabling it to respond effectively to its microenvironment and potentially facilitating critical processes like viral fusion.

### Ectodomain Tilting

The ectodomain constitutes a major part of the HA protein. It is organized around a rigid, trimeric coiled stem region, and prior to activation extends approximately 14 nm outward from the viral surface. The ectodomain base is formed by the so-called 160-helices, which are oriented perpendicularly relative to the molecule’s longitudinal axis.^11^ They do not interact directly with the viral membrane but are connected to the TMD by 10 residue-long, flexible linkers. It has long been known that the ectodomain needs to exhibit a considerable tilt in order to fit into the narrowing space between viral and endosomal membranes during their fusion.^11,84^ The tilt is primarily achieved owing to the flexibility of the linker structures; however, it remains unclear whether it necessitates also any TMD rearrangement.

**Figure 6:**
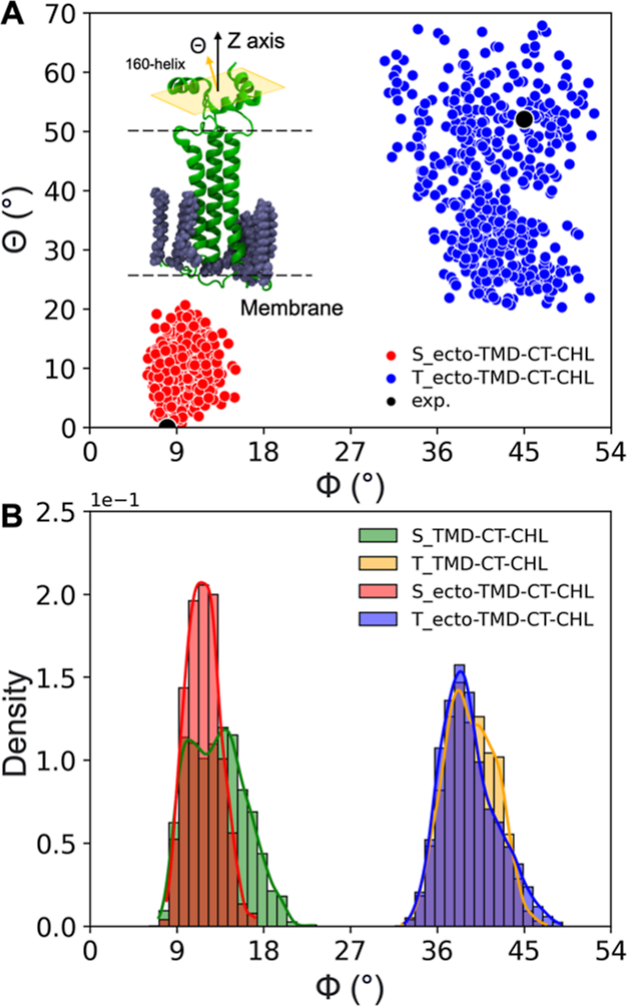
(A) Distribution of TMD and ectodomain tilt angles, ϕ and θ, respectively, for both S (red circles) and T (blue circles) TMD states. (B) Distributions of the ϕ angle for simulations with and without the ectodomain fragment. The ϕ and θ angle values were collected during the last 1 μs of simulation times.

In order to explore the potential coupling between the TMD geometry and the preferred orientation of the ectodomain axis, we performed simulations of systems consisting of 160-helices connected by linkers to membrane-bound TMD-CT in its S and T states, respectively (see Materials and Methods for system details). Each variant was simulated in 5 independent MD runs. Intriguingly, although all 10 simulations were started with the ectodomain axis modeled as perpendicular to the membrane plane, its tilting was observed exclusively in systems, in which the TMD was in the T state (Supporting Information Figure S7). Consequently, upon reaching equilibrium, the simulations sampled two distinct populations (Figure 6A): one, corresponding to S-TMD with a relatively straight ectodomain, and the second with T-TMD and ectodomain tilt with respect to membrane normal by an angle, θ, up to 70°. It is noteworthy that the obtained distributions of TMD and ectodomain tilt angles, ϕ and θ, respectively, are consistent with two alternative geometries captured in cryo-EM data for full-length HA.^11^ Compared to unrestrained MD simulations of isolated TMD-CT in a viral-like membrane, the runs in which the ectodomain base and linkers were added produced somewhat narrower distributions of TMD tilt angles for the S TMD state (Figure 6B), indicating a slight stabilizing effect of the extended HA ectodomain on its membrane anchor. In contrast, in the T TMD case, such effect was not apparent.

### Cytoplasmic Tail Conformation

The determination of the conformational characteristics of the CT region poses a significant challenge due to its close association with the membrane, which limits the feasibility of conventional experimental approaches. In addition, the lack of sequence features indicative of any distinct secondary structure together with a high ratio of post-translational modifications, involving 3 out of 11 residues, complicates further the reasoning based on bioinformatic methods. As a consequence, the specific conformation adopted by CT remains elusive. Nevertheless, some evidence, primarily derived from studies focusing on the isolated CT, suggests the presence of an antiparallel beta structure.^46,85^ Notably, such a structure appears to arise from an inherent conformational propensity of the CT rather than from a tendency for nonspecific ”amyloid-like” aggregation.^85^

Our simulations provide an opportunity for detailed insights into CT in its native-like state, including the palmitoylation of cystein residues, a trimeric arrangement in the presence of TMD, and viral-like membrane. The simulations were carried out for 3 μs and were performed separately for both straight and tilted TMDs. All starting CT conformations correspond to an elongated geometry.

**Figure 7:**
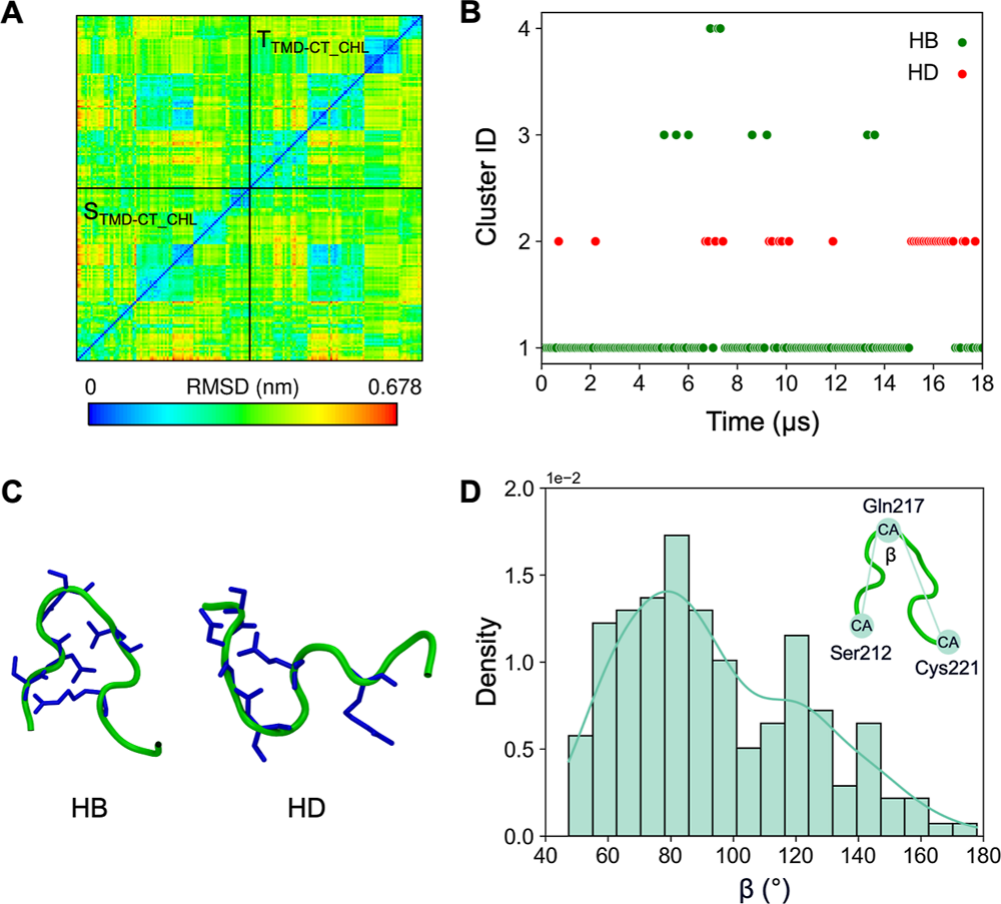
(A) Two-dimensional root-mean-square deviation (2D cross-RMSD) for CT Cα atoms. (B) MD clustering of CT conformations for both TMD configurations. HB represents bent structures stabilized by internal hydrogen bonds and HD represents straight geometries stabilized by hydrophobic interactions. (C) Representative CT structures (medioids) for two main conformation clusters. (D) Distribution of the β angle (see Methods for definition) for CT in both TMD configurations.

As illustrated by the cross-RMSD matrices obtained for Cα atoms (Figure 7A), the conformational ensembles sampled by CT in both TMD configurations were found to be similar. In both scenarios, CT continuously visited different states, and no long-term stabilization was observed throughout the entire MD runs. The clustering and the analysis of the β angle indicative of the CT opening degree (Figure 7B–D) revealed two general families of conformations: a predominant bent structure, β ≈ 80°, and a minor fraction of intrinsically disordered conformations with β > 100°. The bent structures constituted approximately 90% of all CT states observed during the MD simulations. Their existence is consistent with the presence of a highly conserved glycine residue (Gly214), widely recognized as a hallmark of the bent conformation. On the one hand, it confers flexibility to the polypeptide chain and, on the other hand, facilitates the formation of internal, though not necessarily specific, hydrogen bonds, thereby promoting the bent shape. The remaining ∼10% fraction of CT conformations represented an extended configuration, likely stabilized by hydrophobic interactions with a lipid bilayer. Although less prevalent, these disordered conformations contribute to the overall adaptability of CT, potentially enabling transient interactions with various binding targets.

## Conclusions

Our study aimed to gain a deeper understanding of the structural and functional characteristics of the trimeric HA C-terminal part. Its major component, which constitutes the membrane anchor for the entire HA protein, reveals typical sequence features for protein TMDs. Its unique character manifests itself in the ability to adopt two metastable configurations: the ”straight” (S) state with parallel arrangement of the three TMD α-helices, and the ”tilted” (T) state with a noticeable rotational shift of the helical axes. The key interactions enabling such conformational plasticity appear to be provided by Tyr190 side chains. In the S state, their hydroxyl groups form stable hydrogen bonds with hydroxyl groups of Thr192 from adjacent helices. In order to stabilize the T state, they rotate toward the membrane core and engage in hydrogen bonding interactions with buried Ser196. This rearrangement occurs symmetrically at all three interfaces between TMD helices, and its relevance is further underscored by the high degree of sequence conservation of the interacting residues.

An indirect yet important role for the functioning of the above mechanism is played by HA CT, which extends past TMD at the internal side of the viral membrane. It was found to act as a molecular anchor, firmly securing the C-terminal TMD residues at the membrane–water interface, thereby causing a deeper burial of the N-terminal TMD part within the bilayer. Possibly, the fulfillment of this role is the reason for post-translational palmitoylation of CT cysteines, which aid in their tight membrane association. Overall, the anchoring of the TMD C-terminus was found to be essential for the stabilization of hydrogen bonds between Tyr190 and Ser196, likely by their shielding from aqueous solvent. As a consequence, the presence of CT was found to be essential for TMD ability to adopt a fully tilted configuration.

The simulations demonstrated that the inclusion of CHL in the membrane and its associated increase in membrane thickness led to the appearance of a free-energy barrier between the two TMD configurations. Accordingly, the presence of CHL supports a bimodal TMD behavior, in contrast to a continuum of states between S and T extremes observed in the CHL-free case. This effect might be of importance for the positioning of the HA ectodomain with respect to the viral membrane. This hypothesis is supported by the observation of a strong coupling between the TMD configuration and the orientational behavior of the ectodomain part. Our simulations indicated a preference for a straight ectodomain orientation when attached to the TMD in the S configuration and a significant tilt when linked with the TMD in the T state. The observed dependency is consistent with two alternative geometries previously reported on the basis of cryo-EM data. We note that each such geometry must have been represented by a significant fraction of microscopic structures to warrant detection, hence confirming two states rather than continuous HA tilting.

Our results shed light on the conformational behavior of the CT. Regardless of the TMD state, it was found to exhibit a high level of flexibility with no single, unique structure. Roughly 90% of observed configurations corresponded to the bent geometry, achieved due to the presence of a conserved Gly214 and stabilized by internal hydrogen bonds. The remaining 10% of the conformations represented extended configurations, potentially stabilized by hydrophobic interactions with the lipid bilayer.

To sum up, our study indicates remarkable plasticity of the HA membrane anchor. Possibly, it evolved to enable keeping the ectodomain in an extended state in search for the target membrane while also allowing its significant tilt such that it fits into the narrowing space between viral and endosomal membranes during their fusion. Intriguingly, this bimodal TMD behavior seems to be provided by the seemingly inert CT fragment, which turned out to secure proper burial of interhelical hydrogen-bonding switch within the membrane, thus enhancing its stability. It remains to be validated whether the targeted perturbation of this mechanism might impede HA orientation changes required for membrane fusion, thus potentially neutralizing virus infectivity.

## Data Availability

The original contributions presented in the study are publicly available. These data can be found at the Figshare repository under the following URL: 10.6084/m9.figshare.24259978.
