# Supplementary material for: Molecular Mechanisms behind Conformational Transitions of the Influenza Virus Hemagglutinin Membrane Anchor
Source: J Phys Chem B. 2023 Oct 25;127(44):9450–60. doi: 10.1021/acs.jpcb.3c05257 (PMC10641832; doi:10.1021/acs.jpcb.3c05257)
Supplement: Supplementary file 1 — jp3c05257_si_001.pdf [file jp3c05257_si_001.pdf]

Electronic Supporting Information for:

Molecular Mechanisms behind Conformational Transitions of Influenza Virus Hemagglutinin Membrane Anchor

Michal Michalski [1)], Piotr Setny [1)]

[1)] Centre of New Technologies, University of Warsaw, 02-097, Warsaw, Poland

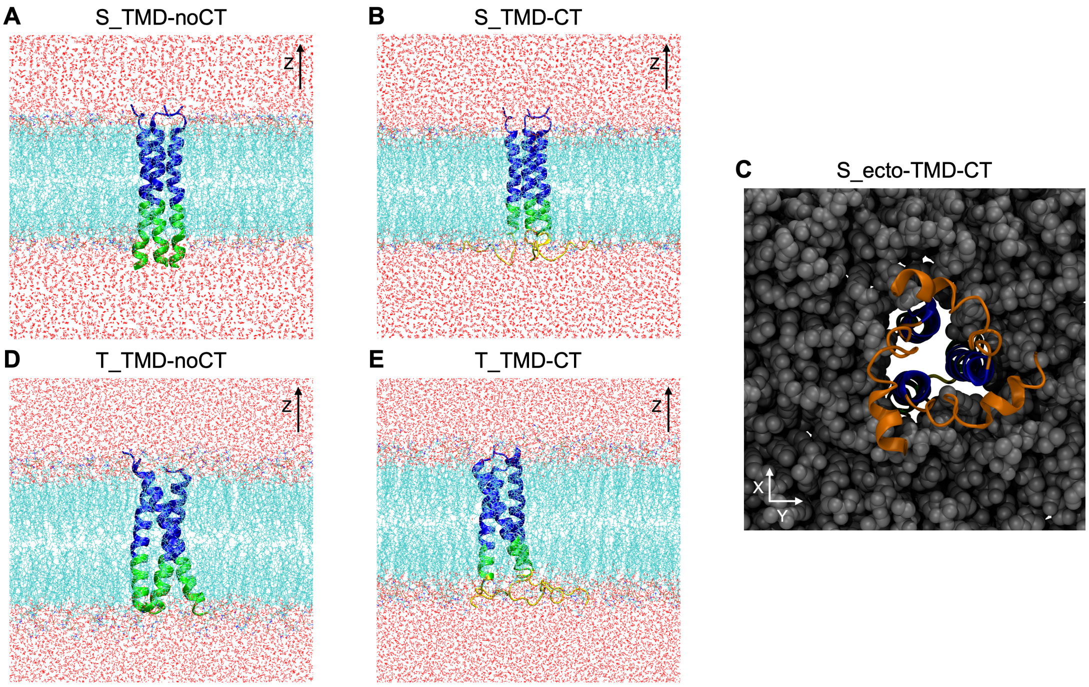

Figure S1. Starting straight (A, B, C) and tilted (D, E) TMD configurations for 3 μs production runs of membrane-bound C-terminal HA2 trimers. The structures representing HA2 ectodomain and N-terminal TMD part (orange, blue ribbons) were obtained from PDB ids 6HJQ and 6HJR. Missing HA2 C-terminus containing TMD (green ribbon) and CT (yellow ribbon) parts were reconstructed using the BIOVIA Discovery Studio 2021. Lipid and water molecules shown as lines. Fatty acids are not shown for clarity.

Table S1. Harmonic force constants and distances applied to the 160-helix domain and used in MD simulations of trimeric C-terminus within lipid bilayer environment.

Pairwise restraint interaction	Force constant (kJ mol^{-1}nm^{-2})	Reference distance (nm)
Ser166:A:Cα $\cdots$ Ser166:B:Cα	1000	2.572
Ser166:A:Cα $\cdots$ Ser166:C:Cα	1000	2.572
Ser166:B:Cα $\cdots$ Ser166:C:Cα	1000	2.572
Ser166:A:Cα $\cdots$ Asp177:C:Cα	1000	1.057
Ser166:B:Cα $\cdots$ Asp177:A:Cα	1000	1.057
Ser166:C:Cα $\cdots$ Asp177:B:Cα	1000	1.057
Asp177:A:Cα $\cdots$ Asp177:B:Cα	1000	1.556
Asp177:A:Cα $\cdots$ Asp177:C:Cα	1000	1.556
Asp177:B:Cα $\cdots$ Asp177:C:Cα	1000	1.556

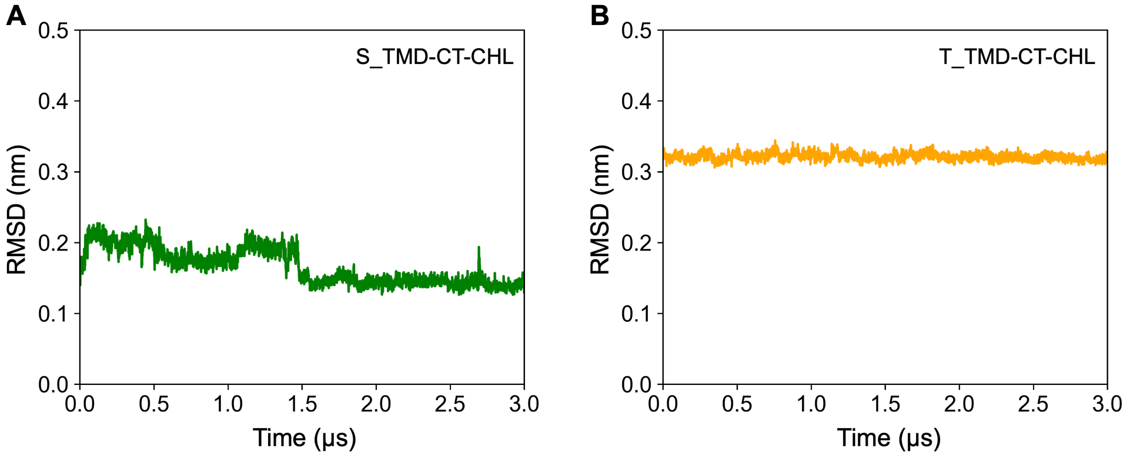

Figure S2. Time evolution of root-mean-square deviation of atomic positions (RMSD) during straight (A) and tilted (B) TMD-CT-CHL simulations. The RMSD was calculated for TMD Cα atoms between MD and cryo-EM (PDB: 6HJQ, 6HJR) structures as reference.

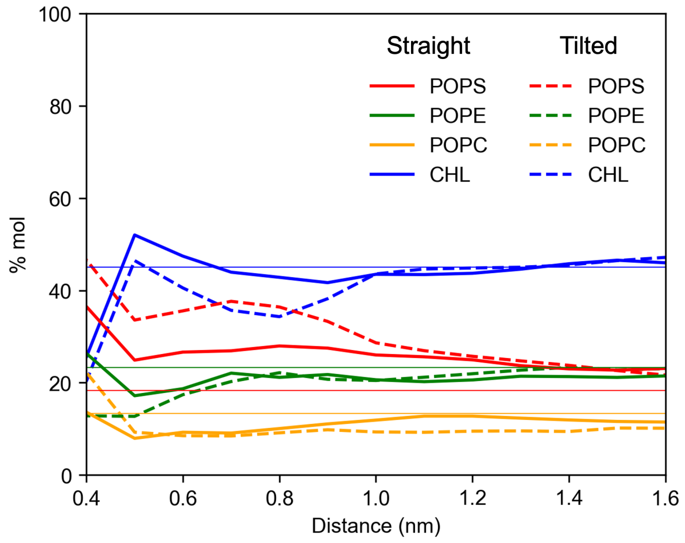

Figure S3. Lipid molar fractions as a function of distance from HA2 C-terminus. The distance is calculated as a minimal distance in XY plane (i.e membrane plane) between lipid phosphate or CHL hydroxyl group oxygen atoms and the nearest Cα protein atom. The profiles were obtained from the last 2 μs of unrestrained MD runs with CHL molecules for both S and T TMD states. Solid horizontal lines correspond to reference lipid concentrations.

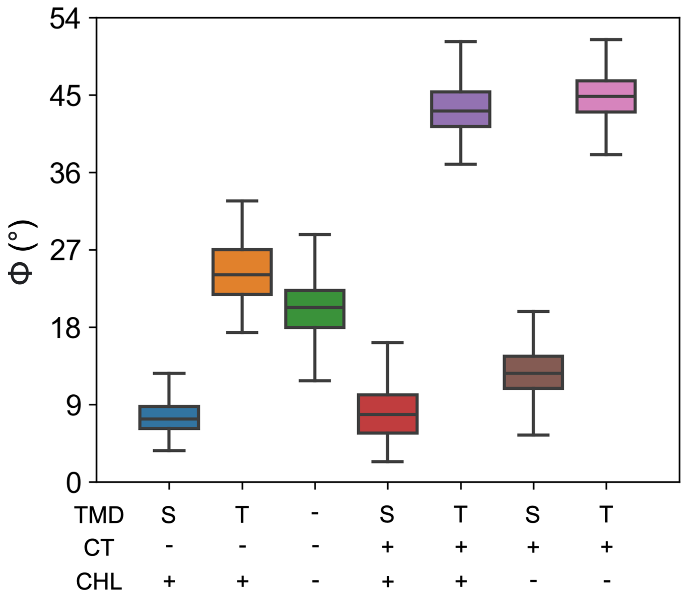

Figure S4. Distribution of TMD helices tilt angles obtained from unrestrained 3 μs MD simulations for all studied cases. The ϕ values were obtained from the last 2 μs of MD runs.

Table S2. Hydrophobic length obtained from 3 MD snapshots for both straight and tilted TMD states.

Simulation System	Simulation snapshot	Hydrophobic length (nm)
	2.0 μs	3.56 nm ± 0.19
S_TMD-CT-CHL	2.5 μs	3.58 nm ± 0.18
	3.0 μs	3.58 nm ± 0.11
	2.0 μs	3.52 nm ± 0.16
T_TMD-CT-CHL	2.5 μs	3.58 nm ± 0.09
	3.0 μs	3.56 nm ± 0.21

Table S3. P-P and hydrophobic thickness obtained through 300 ns MD simulation, conducted using two setups representing protein-free influenza virus lipid membrane composition.

Membrane composition	Composition ratio	P-P thickness (nm)	Hydrophobic thickness (nm)
POPC:POPE:POPS	52:108:80	4.25	3.16
POPC:POPE:POPS:CHL	32:56:44:108	4.50	3.60

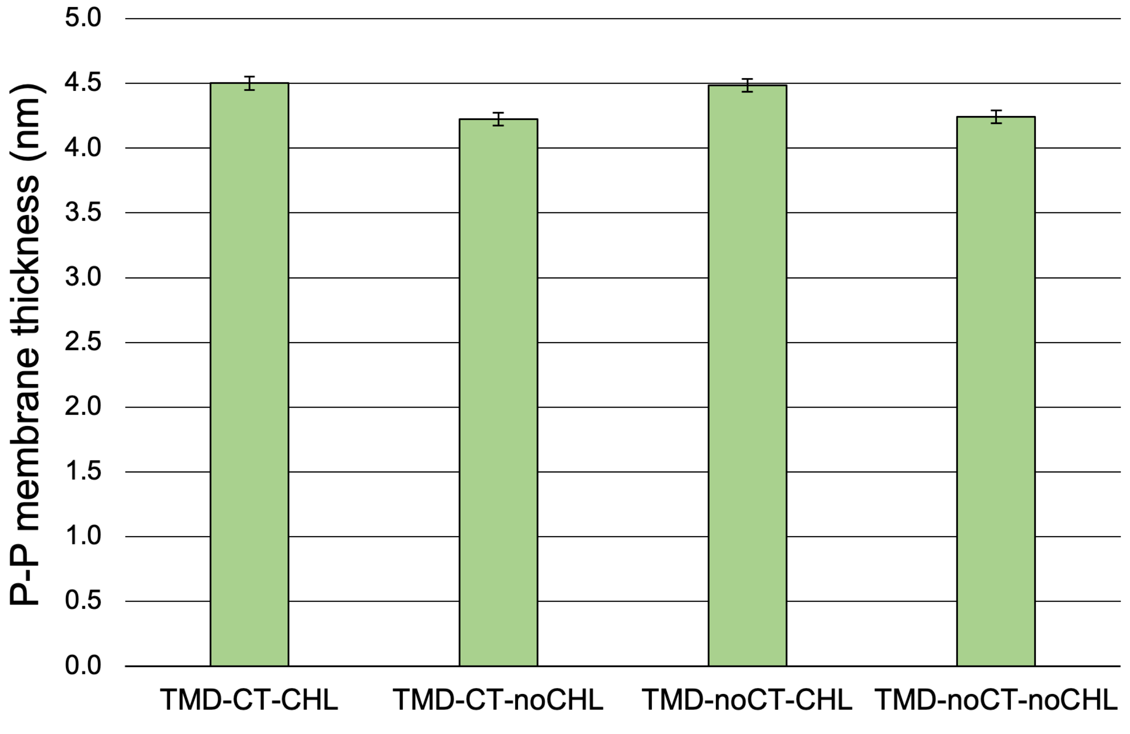

Figure S5. The P-P membrane thickness, calculated as distance between center of mass of phosphate atoms in upper and lower leaflets, for $\phi \sim 10^\circ$. Error bars correspond to standard deviations.

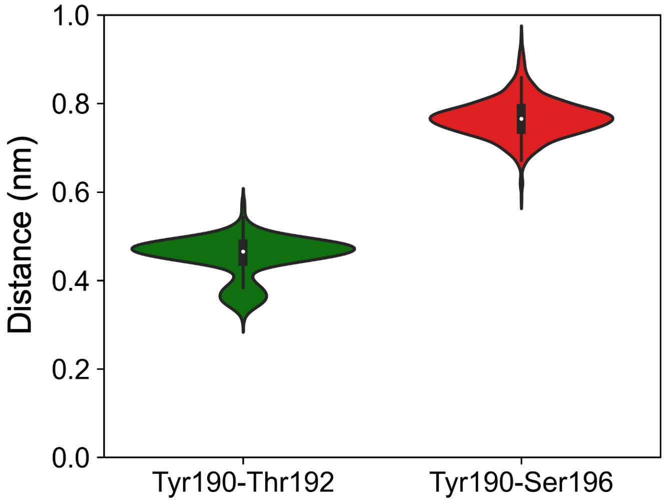

Figure S6. The donor-acceptor mean distances for hydrogen bonds at $\phi \sim 18°$ calculated between Tyr190:OH$\cdots$Thr192:OG1 and Tyr190:OH$\cdots$Ser196:OG atoms.

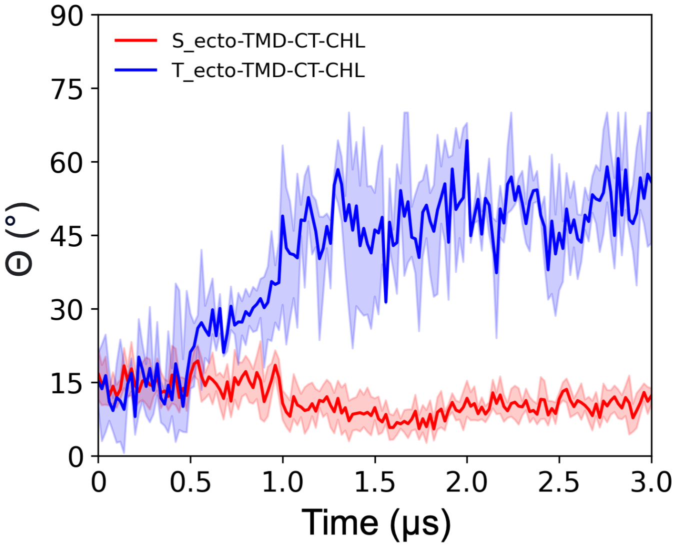

Figure S7. Time evolution of ectodomain tilt angle, θ, during ectodomain-TMD-CT simulations. Shadows correspond to one standard deviation across 5 MD runs for both S and T states, respectively.
